# Facile Post Treatment of Ag Nanowire/Polymer Composites for Flexible Transparent Electrodes and Thin Film Heaters

**DOI:** 10.3390/polym13040586

**Published:** 2021-02-15

**Authors:** In Su Jin, Hee Dong Lee, Seok Il Hong, Woosung Lee, Jae Woong Jung

**Affiliations:** 1 Department of Advanced Materials Engineering for Information and Electronics, Integrated Education Institute for Frontier Materials (BK21 Four), Kyung Hee University, 1732 Deogyeong-daero, Giheung-gu, Yongin-si 446-701, Gyeonggi-do, Korea; jininsu1022@gmail.com; 2 Advanced Textile R&D Department, Korea Institute of Industrial Technology (KITECH), Ansan 15588, Korea; lhd0121@kitech.re.kr (H.D.L.); redstone@kitech.re.kr (S.I.H.)

**Keywords:** silver nanowires, transparent conductive electrode, film heaters

## Abstract

Typical polyol-based synthesis of silver nanowire employs insulating polymer as a surfactant for the silver nanowire growth, which limits direct contact between each nanowire and thus its optoelectronic properties. We herein demonstrate that a simple solvent treatment effectively removes the insulating polymer around Ag NWs, leading to significantly decreased sheet resistance (~12 Ω/sq) with an increased transmittance (81% @ *T*_550_), as compared to other post-treatments. We successfully demonstrate the transparent film heaters using the solvent-treated Ag NWs network, which rapidly exhibited 150 °C under a bias of 5 V. Flexible film heaters on plastic substrate is also demonstrated, suggesting a great potential of the solvent treatment process of Ag NWs for flexible transparent electrode and film heater applications.

## 1. Introduction

A transparent conductive electrode (TCE) is an essential element in optoelectronic devices such as organic light-emitting diodes (OLEDs), liquid crystal displays (LCD), e-papers, touch screen panels, sensors, and solar cells [1,2,3,4,5,6,7]. Indium tin oxide (ITO) is the most well-known TCE material due to its excellent electrical conductivity and optical transmittance. However, low thermal response, brittleness under bending or stretching, low chemical stability, and high vacuum/temperature processing hinder further applications of ITO in flexible or stretchable devices [8]. In addition, high material cost of ITO is another major hurdle for fabrication of large-area optoelectronic devices. Recently, several alternatives for TCE such as graphene [9], single walled carbon nanotube [10], and conducting polymers have been studied as a replacement of ITO [11]. Although those have been reported comparable optoelectronic properties of TCE in terms of electrical conductivity and optical transmittance to ITO [12], there still remain limitations in reproducibility, solution processing at low temperature, and materials cost.

Metallic nanomaterials have emerged as promising candidates for replacement of ITO due to their native metallic conductivities [13]. Amongst, nanowires is a one-dimensional nanostructure that is advantageous for charge transfer on a flat substrate in a variety of optoelectronic devices over other nanostructures, such as spherical or cubical nanoparticles. Specifically, silver nanowires (Ag NWs) have been vigorously studied because its thin film can be prepared from a dispersion at room temperature. Moreover, mechanical compliance and flexibility have leaded to successful demonstration of optoelectronic devices such as OLEDs, touch screen, and organic solar cell, in recent years [14,15,16,17]. Several synthetic protocols have also been developed to control the quality of Ag NWs such as length, diameter, morphology. The most well-known procedure for preparation of Ag NWs is polyol synthesis in which polyvinylpyrrolidone (PVP) promotes not only the growth of Ag crystals in one dimension, but also the dispersion in organic medium [18,19,20]. However, the insulating nature of PVP inhibits electrical conduction across the wires, which increases the resistivity of the Ag NWs network. In this regard, the removal of PVP surfactant is challenging to reduce the contact resistance between Ag NWs. In the last 4 years, several post treatments such as thermal, mechanical, and plasmonic processes have been proposed for effective removal of the un-desired surfactants of the Ag NWs network [12,21,22,23,24,25]. Most of these methods, however, usually require a complicated process using specialized instruments or high-temperature annealing steps, which are not compatible with cost-effective and large area processing on flexible substrates.

In this paper, we suggest a simple and facile method for achieving high optoelectronic properties of Ag NWs network to be used in TCE. In order to improve the electrical and the optical performance, the influence of the post treatments on the properties of Ag NWs network was investigated. The pristine Ag NWs networks were treated for three different ways: (i) Thermal annealing; (ii) solvent treatment (dipping in ethanol); (iii) N_2_-gas blowing. From the morphology observation with elemental analysis, it was found that the solvent treatment is the most effective method to remove the surfactant of Ag NW, which lead to improvement of sheet resistance (*R*_sh_) and optical transmittance of the Ag NWs network. Considering that the ethanol treatment is a non-toxic process suitable to a flexible substrate, we demonstrated the flexible thin film heaters (TFHs) with a rapid heating at lower operation voltage based on high quality Ag NWs network. This work clearly demonstrates a feasibility of solvent treatment in the fabrication of high-quality Ag NWs network for preparation of high performance TCE.

## 2. Materials and Methods

### 2.1. Materials

AgNO_3_ (99%), ethylene glycol (99.5%), MnCl_2_, (99.0%), KBr (99%), and PVP (M_w_ = 55,000 g/mol) were provided from commercial sources (Sigma-Aldrich, St. Louis, MI, USA and Acros Organics, Geel, Belgium), and all the materials were used as received without further purification.

### 2.2. Controlled Synthesis of Ag NWs

Ag NWs were synthesized via typical polyol synthesis as reported elsewhere [18,19,20]. Briefly, 1.5 molar ratio of PVP was dissolved in 10mL of ethylene glycol. After the temperature of the mixture was stabilized at 100 °C, AgNO_3_ precursor was added slowly into the mixture. MnCl_2_ and KBr were subsequently added to the solution, while the mixture was stirred at 120 °C. The diameter of Ag NWs was controlled by using different concentrations of bromide in the solution (3 mM and 8 mM for thin and thick Ag NWs) and KBr (1 mM for thin Ag NWs, and 2 mM for thick Ag NWs). The reaction mixture was allowed to react for a further 8 h and cooled to room temperature. The nanowires were washed with ethanol and collected by centrifugation at 4000 rpm for 30 min. This was repeated three times to remove the residual solvent and surfactant.

### 2.3. Fabrication and Post Treatment of Ag NWs Network

Glass substrates were cleaned by ultrasonic in deionized water, acetone, and isopropyl alcohol for 15 min. In order to obtain a hydrophilic surface, the cleaned substrate was treated with UV-O_3_ treatment for 15 min. Two different Ag NWs solutions with different diameter were spin coated onto glass substrate. Before spin coating, the Ag NWs solution was diluted in isopropyl alcohol to the desired concentration. The coverage of the Ag NWs network on the substrate was varied by changing the spinning rate to control the optical and electrical properties. Post treatments of the Ag NWs network were conducted by four three different types: (i) Thermal annealing at 130 °C for 20 min for thermal treatment, (ii) dipping in ethanol for 5 min for solvent treatment; (iii) N_2_ (99.9%) gas-blowing for 10 s for mechanical treatment. The thin film heater (20 × 20 mm^2^) was demonstrated using a two terminal side contact electrodes which are composed of a silver layer (100 nm) deposited by thermal evaporation in vacuum chamber, and then Ag paste.

### 2.4. Characterizations

The sheet resistance of Ag NWs network was measured by a four-point probe (Ossila). The surface of Ag NWs network was observed by scanning electron microscopy (S-4800, Hitach) and atomic force microscope (CoreAFM, Nanosurf, Liestal, Switzerland). Optical transmittance of Ag NWs network was measured by UV-Vis spectrophotometer (Cary 100, Agilent, Santa Clara, CA, USA). For investigating the thin film heater, the DC voltage was supplied by a power supply (Keithley 2400, Keithley, Cleveland, OH, USA) to the Ag NWs network through a silver contact electrode. The temperature variation of the Ag NWs network was measured by using an infrared thermal camera (ETS320, FLIR, Wilsonville, OR, USA).

## 3. Results

**Figure 1:**
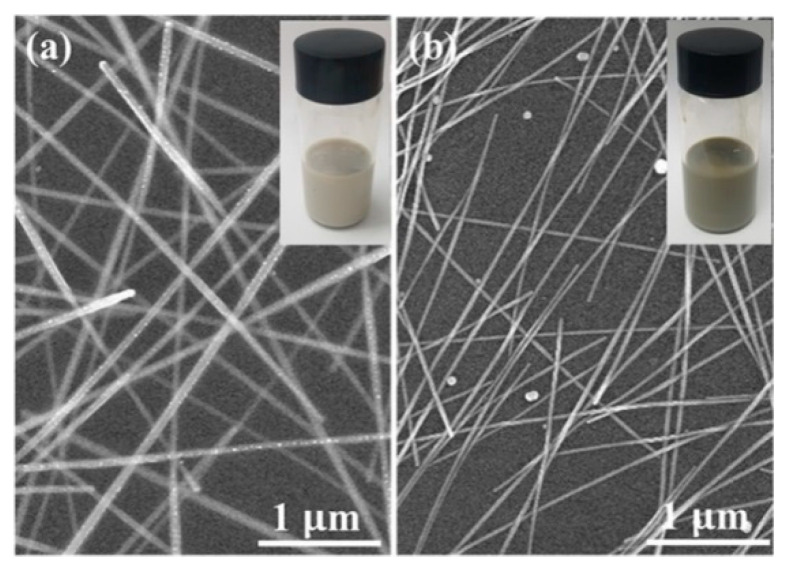
(**a**) SEM image of a thick Ag NWs network and (**b**) a thin Ag NWs network. Inset is a Ag NWs solution with thin and thick diameter.

Ag NWs were synthesized by the polyol synthetic protocols through the reduction of AgNO_3_ in the presence of PVP as a typical surfactant for the NW growth [18]. The as-synthesized Ag NWs were purified by consecutive centrifugation. It has been reported that the length and diameter of Ag NWs varied drastically with the molar ratio of reagents, reaction temperature, and the stirring speed [22,23]. During the synthesis and growth of Ag NWs, the PVP capping agent was gradually adsorbed on the surface of the silver crystals, and the very thin PVP layer formed on top of the crystals, formed from the merging of the crystals. The PVP layer prevents the agglomeration of Ag NWs due to steric repulsion [24], while a too thick layer also increases the contact resistance. As a result, two Ag NWs with different diameters were prepared by appropriate tailoring of the ratio of PVP during the growth of Ag NWs, as displayed in Figure 1. Each Ag NWs possesses a diameter of ~65 nm and ~25 nm, thus denoted as thick and thin Ag NWs hereafter, respectively. As seen in inset images of Figure 1, the as-synthesized solutions of Ag NWs dispersed in isopropyl alcohol exhibit slightly different colors at the same concentration: Light grey solution for thick Ag NWs; dark grey solution for thin Ag NWs. Thin films of Ag NWs networks were fabricated on glass substrate using a spin-coating by changing the spinning rate to control the film thickness. (Figures S1–S3) In consideration of the limitation of *R*_sh_ required for TCEs in optoelectronic devices, we investigated the optoelectronic properties of the Ag NWs in the range of *R*_sh_ < 15 Ω/sq in this work.

**Figure 2:**
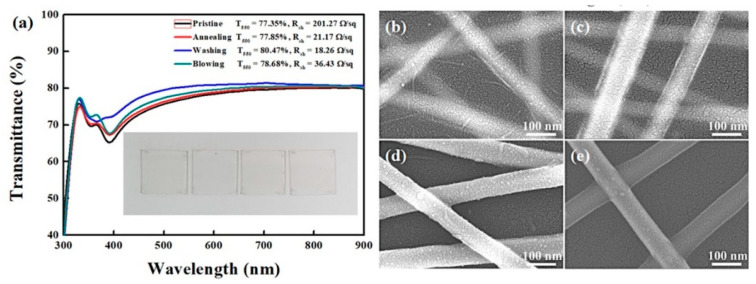
(**a**) Optical transmittance and sheet resistance of thick Ag NWs network with various post treatment. Inset is a photograph of a thick Ag NWs network. (**b**–**e**) SEM images of a thick Ag NWs network followed by different post-treatment. (**b**) SEM images of the pristine thick Ag NWs network, (**c**) thick Ag NWs network after thermal annealing at 130 °C for 20 min, (**d**) thick Ag NWs network after solvent dipping, and (**e**) thick Ag NWs after N_2_ gas-blowing.

**Figure 3:**
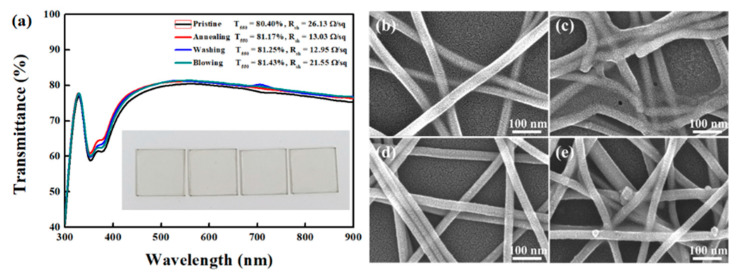
(**a**) Optical transmittance and sheet resistance of a thin Ag NWs network with various post treatment. Inset is a photograph of the thin Ag NWs network. (**b**–**e**) SEM images of the thin Ag NWs network followed by different post-treatment. (**b**) SEM images of the pristine thin Ag NWs network, (**c**) thin Ag NWs network after thermal annealing at 130 °C for 20 min, (**d**) thin Ag NWs network after solvent dipping, and (**e**) thin Ag NWs after N_2_ gas-blowing.

Figure 2 shows the thick Ag NWs network films with different post-treatments. A variety of post-processing methods, such as heating and pressing, plasma treatments have been proposed to thin down the PVP layer [12,22,26]. However, it is feasible approach to use the solvent treatments to remove the PVP capping layer on Ag NWs because PVP is soluble in various solvents such as water, methanol, ethanol, and acetic acid [24]. We chose an ethanol as a treatment solvent owing to its low toxicity, low price, and low boiling point. Ethanol dipping would be effective to wash out the PVP capping layer, because the PVP on Ag NWs was adsorbed through weak van der Waals force, as confirmed by no specific absorption of PVP (3420 cm^−1^ or 2950 cm^−1^) from Fourier-transform infrared spectroscopy (Figure S4). The transmittance of Ag NWs network was slightly improved after post-treatments. In particular, the solvent treatment resulted in improvements from 201.27 to 18.21 Ω/sq and 77.35 to 80.47% for *R*_sh_ and *T*_550_, respectively. Figure 2b–e displays the SEM images of thick Ag NWs networks with different treatments (High magnification SEM images were provided in Figure S5.). The blurred edge of pristine Ag NWs network became clear after the solvent treatment, which is far different to the thermal annealing, indicating the effectiveness of the solvent treatment to reduce the PVP layer. The partial removal of the insulated PVP layer or soldering of each NW from the Ag NWs surface is expected by thermal annealing to result in an improved electrical connection between the Ag NWs. However, additional N_2_ blowing does not change significantly. For the case of thin Ag NWs, *R*_sh_ was much lower as compared to that of thick Ag NWs network (Figure 3) (High magnification SEM images were provided in Figure S6.). The best result was observed from the solvent treatment (12.85 Ω/sq and 81.25% for *R*_sh_ and *T*_550_, respectively), which highlights the efficacy of the solvent treatment for removal of PVP capping layer on Ag NWs. It is interesting that thin Ag NWs network possesses better *R*_sh_ and *T*_550_ than those of the thick Ag NWs network in the same post treatment. This could be attributed to the higher density and resulting higher coverage of thin Ag NWs network relative to the thick Ag NWs, as displayed in Figures S7 and S8. The Ag NWs network prepared in this work also showed high stability with low deviation upon subsequent measurement, as seen in Figure S9.

It is very challenging to fabricate a TCE with low resistance and high transmittance simultaneously because of the inverse proportional relationship between optical transmittance and electrical conductivity. The figure of merit (FoM), which is a parameter that characterizes the relationship between transmittance and conductivity in a TCE, can be derived as

(1) $$\frac{\sigma_{dc}}{\sigma_{opt}}={\left\{\left(T^{-\frac{1}{2}}-1\right)\frac{2R_{sh}}{Z_{0}}\right\}}^{-1}$$

where σ*_dc_* is the DC conductivity of the film, *σ_opt_* is the optical conductivity at *λ* nm, *T* is the transmittance at *λ* nm, *R*_sh_ is the sheet resistance, and *Z*_0_ is impedance of free space (377 Ω). The FoM value of the Ag NWs network films fabricated in this study increased from 90.20 to 134.09 as the diameter of NW decreases from 65 nm to 25 nm, when we observed that diameter of Ag NWs decreases from 65 nm to 25 nm. The FoM value of the Ag NWs electrode with a thin diameter is higher than the Ag NWs electrode with a thick diameter. In addition, substantial improvement of transmittance and sheet resistance of Ag NWs network after ethanol treatment would originate from the junction resistance decrease due to the reduced PVP layer on each Ag NW. The comparison of this work to other reports of TCE made of Ag NWs with different treatments also indicates that the efficacy of ethanol treatments to enhance the optoelectronic properties of the Ag NWs network (Figure S10).

**Figure 4:**
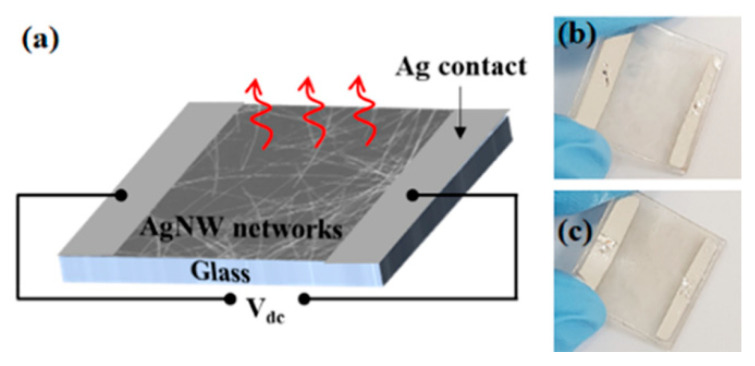
(**a**) Schematic illustration of layouts for a film heater with Ag NWs networks. (**b**,**c**) A photograph of Ag NWs of the thick and thin diameter-based film heaters (size = 20 × 20 mm^2^).

**Figure 5:**
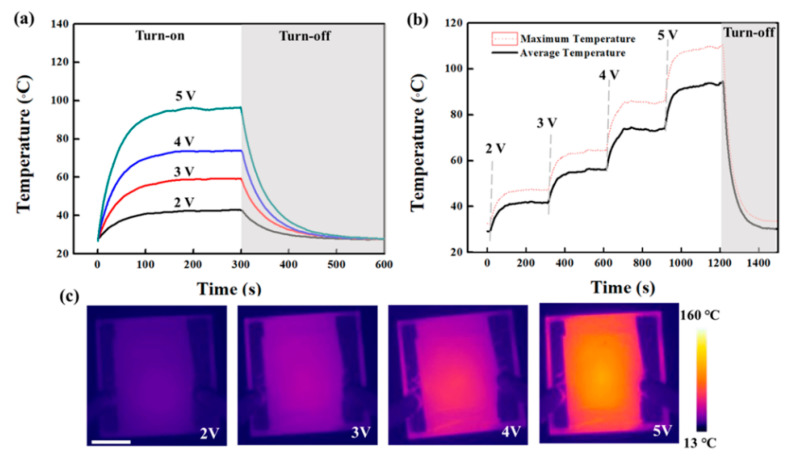
(**a**) Time-dependent temperature of thick Ag NWs network-based film heaters (20 × 20 mm^2^) under operation at different voltages. (**b**) Temperature evolution of thick Ag NWs network-based film heaters at stepwise voltage rise from 2 to 5V. (**c**) Temperature profiled of 20 × 20 mm^2^ electrodes, measured using an IR camera. (scale bar = 6.5 mm).

**Figure 6:**
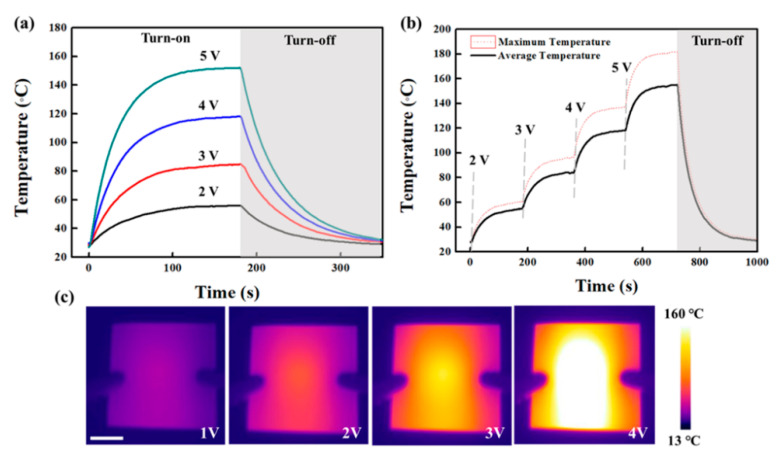
(**a**) Time-dependent temperature of thin Ag NWs network-based film heaters (20 × 20 mm^2^) under operation at different voltages. (**b**) Temperature evolution of thin Ag NWs network-based film heaters at stepwise voltage rise from 2 to 5 V. (**c**) Temperature profiled of 20 × 20 mm^2^ electrodes, measured using an IR camera (scalebar = 6.5 mm).

**Figure 7:**
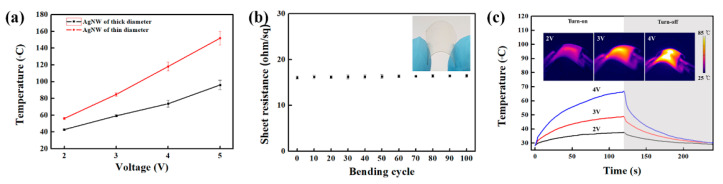
(**a**) The voltage-dependent temperature evolution, (**b**) sheet resistance variation, and (**c**) time-dependent temperature of Ag NWs network-based flexible film heaters at different voltages. The bending test was repeatedly performed to 7 mm bending radius.

In order to demonstrate the potential of a TCE, we fabricated TFHs using a thin Ag NWs network obtained after solvent treatment. Figure 4a shows a photograph of TFHs made of the Ag NWs network with two different diameters. The DC voltage was supplied to the transparent heater via a silver contact, and then the surface temperature of the Ag NWs network was recorded by the IR thermometer. Figure 5a displays the temperature variations of a TFH made of a thick Ag NWs network (*R*_sh_ = 18.21 Ω/sq). As the input voltage increases from 2 to 5 V, the thick Ag NWs network reaches the average temperature of ~95 °C with a maximized temperature of 110 °C. This is a comparable heating capability to those of reports of Ag NWs network-based TFHs for achieving >100 °C [27,28,29,30]. A response time, which is required to reach stable temperature, is one of the key factors for evaluating the performance of the heater. Regardless of the input voltage, the time required to rise to 90% of the average temperature is within 180 s for an input voltage of 2 to 5 V. Figure 5b observed the temperature variation of the TFH by sequential increase of the input voltage. As the voltage increases stepwise from 2 to 5V, the average temperature and maximum temperature quickly stabilized in a short time with a linearly increased temperature at the input voltage. Figure 5c shows a uniform heat distribution with a photograph of the Ag NWs network with a thick diameter-based film heater, along with is a corresponding infrared image. Figure 6a shows the temperature variation of the TFHs fabricated with a thin Ag NWs network (*R*_sh_ = 12.85 Ω/sq). The thin Ag NWs network generated much higher temperatures at each input voltage as compared to the thick Ag NWs network film. This result originates from the improved conductivity of Ag NWs with thin diameter electrodes with the solvent treatment, and resulting in low *R*_sh_ of the Ag NWs network. For the input voltage of 4 V, the average temperature and the maximum temperature were 117 °and 137 °C. In other words, only 3.7 V was needed to reach the average temperature of 100 °C at an area of 20 × 20 mm^2^. In addition, the time required to rise to 90% of the average temperature is within 120 s, much faster than then thick Ag NWs network. Figure 6b shows stabilized temperature rise by increasing the input voltage over time. As the voltage increases stepwise from 2 to 5V, each average temperature over time of the heater is maintained as noted Figure 6a. Figure 6c also shows a uniform heat distribution of thin Ag NWs network, observed by infrared image. These results certainly indicate the efficient conversion of electrical energy to heating at low input voltage by high quality Ag NWs network obtained by solvent treatment. Moreover, the diameter of Ag NWs is critical to determine not only the optoelectronic performance of the TCE, but also a film heating property such as TFHs (Figure 7).

For the practical applications, fast and low voltage operation of flexible TFHs is advantageous, such as thermal patches for pain management. Thus, the highly conductive Ag NWs network were formed on the PET substrate by using the ethanol treatment, as shown in inset of Figure 7b. The flexible TFHs (*R*_sh_ of 16.29 Ω/sq with a *T*_550_ of 80.2%.) exhibited good flexibility, retaining its *R*_sh_ of 16.48 Ω/sq upon consecutive bending test up to 100 cycles. As shown in Figure 7c, the flexible TFHs with a silver contact also work well even in a bent state. The flexible TFHs reached >70 °C for the input voltage of 4 V. The discrepancy of thermal heating capability of Ag NWs network on glass and PET substrate would be originated from the heat-absorbing behavior of PET substrate. An essential thing is that the Ag NWs TFHs work well and are stable under mechanical motion, suggesting a great potential for application in practical flexible TFHs.

## 4. Discussion

In conclusion, we demonstrated a facile method to prepare a high quality Ag NWs network utilizing solvent treatment. Although the post treatments for Ag NWs networks have been studied vigorously so far, the typical thermal or complex processes could not be readily applied to large-scale Ag NWs networks or their commercialization. In this study, the efficacy of solvent treatment (ethanol dipping) for removal of the insulating PVP around the Ag NWs was addressed. It is impressive that the solvent treatment lead to lower *R*_sh_ of the Ag NWs network to ~12 Ω/sq with a transmittance of ~81% (*T*_550_). The highly conductive and transparent Ag NWs network also demonstrated the TFHs with ~150 °C at the input voltage of 5 V. The solvent treatment was also effective on plastic substrate, which resulted in flexible TCE based on thin Ag NWs network with low *R*_sh_ of ~16 Ω/sq and transmittance of ~80% (*T*_550_). The flexible TFHs also showed a promising result with ~70 °C for the input voltage of 4 V, showing the fact that the ethanol dipping is a straightforward and non-toxic process for the preparation of high performance flexible TCEs and TFHs based on Ag NWs network.

## Supplementary Materials

The following are available online at https://www.mdpi.com/2073-4360/13/4/586/s1, Figures S1 and S2: Transmittance of Ag NWs network, Figure S3: Photographic images of Ag NWs network, Figure S4: FT-IR spectra of Ag NWs networks, Figures S5 and S6: SEM images of Ag NWs network, Figures S7 and S8: AFM topographic images of Ag NWs network, Figure S9: Sheet resistance variation, Figure S10: Comparison of sheet resistance reported elsewhere.
