# Supplementary material for: Facile Post Treatment of Ag Nanowire/Polymer Composites for Flexible Transparent Electrodes and Thin Film Heaters
Source: Polymers (Basel). 2021 Feb 15;13(4):586. doi: 10.3390/polym13040586 (PMC7919462; doi:10.3390/polym13040586)
Supplement: Supplementary file 1 [file polymers-13-00586-s001.pdf]

polymers

Supplementary data for

Facile Post Treatment of Ag Nanowire/Polymer Composites for Flexible Transparent Electrodes and Thin Film Heaters

In Su Jin [1], Hee Dong Lee [2], Seok Il Hong [2], Woosung Lee [2,*] and Jae Woong Jung [1,*]

[1] Department of Advanced Materials Engineering for Information and Electronics, and Integrated Education Institute for Frontier Materials (BK21 Four), Kyung Hee University, 1732 Deogyeong-daero, Giheung-gu, Yongin-si, Gyeonggi-do 446-701, Korea; jininsu1022@gmail.com
[2] Advanced Textile R&D Department, Korea Institute of Industrial Technology (KITECH), Ansan 15588, Korea; lhd0121@kitech.re.kr (H.D.L.); redstone@kitech.re.kr (S.I.H.)
* Correspondence: wslee@kitech.re.kr (W.L.); wodndwjd@khu.ac.kr (J.W.J.)

Citation: Jin, I.S.; Lee, H.D.; Hong, S.I.; Lee, W.; Jung, J.W. Facile Post Treatment of Ag Nanowire/Polymer Composites for Flexible Transparent Electrodes and Thin Film Heaters. *Polymers* **2021**, *13*, 586. https://doi.org/10.3390/polym13040586

Academic Editor: Nektaria Marianthi Barkoula
Received: 28 January 2021
Accepted: 12 February 2021
Published: 15 February 2021

Publisher's Note: MDPI stays neutral with regard to jurisdictional claims in published maps and institutional affiliations.

Copyright: © 2021 by the authors. Licensee MDPI, Basel, Switzerland. This article is an open access article distributed under the terms and conditions of the Creative Commons Attribution (CC BY) license (http://creativecommons.org/licenses/by/4.0/).

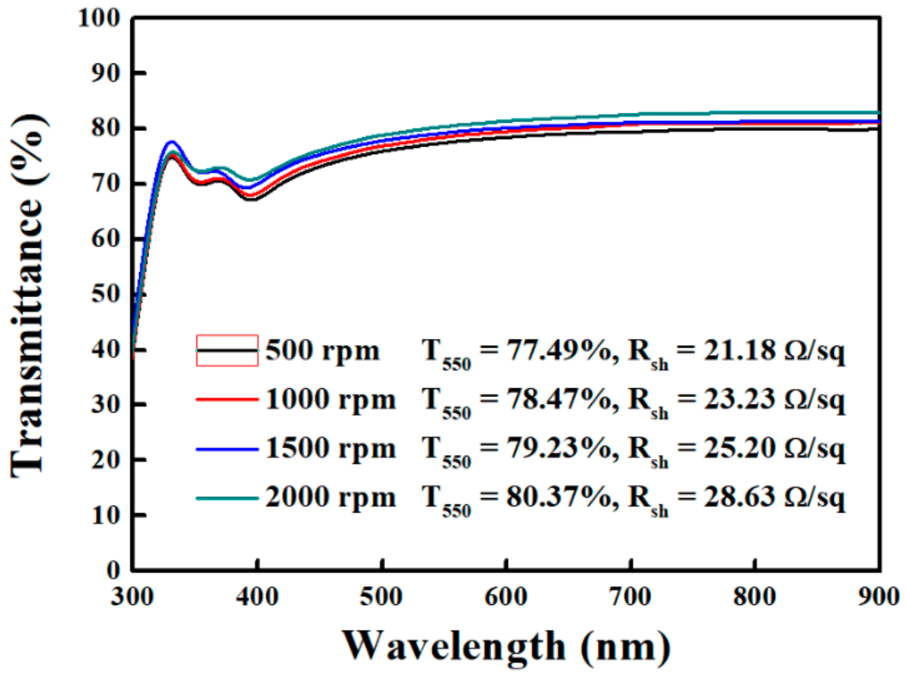

Figure S1. Transmittance of thick Ag NWs network at various spin rate.

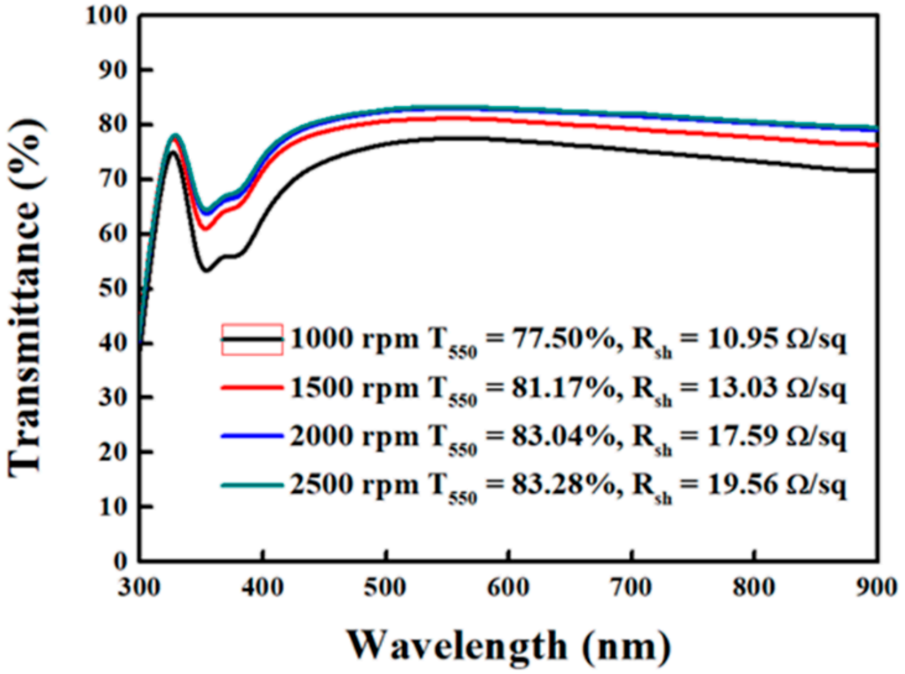

Figure S2. Transmittance of thin Ag NWs network at various spin rate.

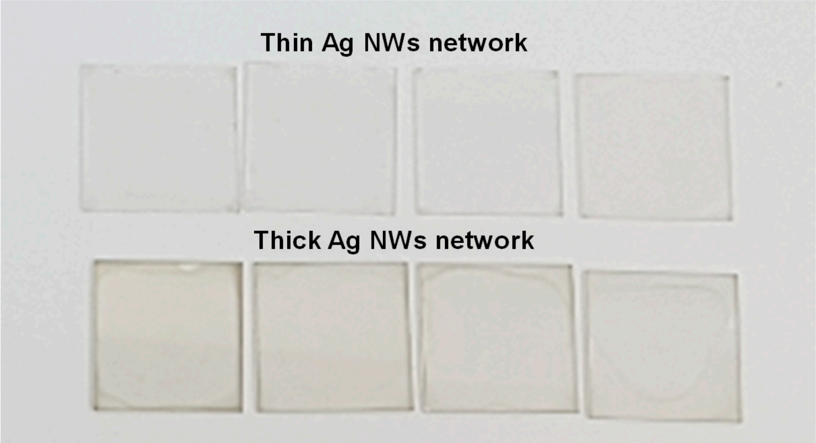

Figure S3. Photographic images of AgNWs network with the thick and thin diameter at the various spin coating speed (left to right: 1000 rpm, 1500 rpm, 2000 rpm, 2500 rpm).

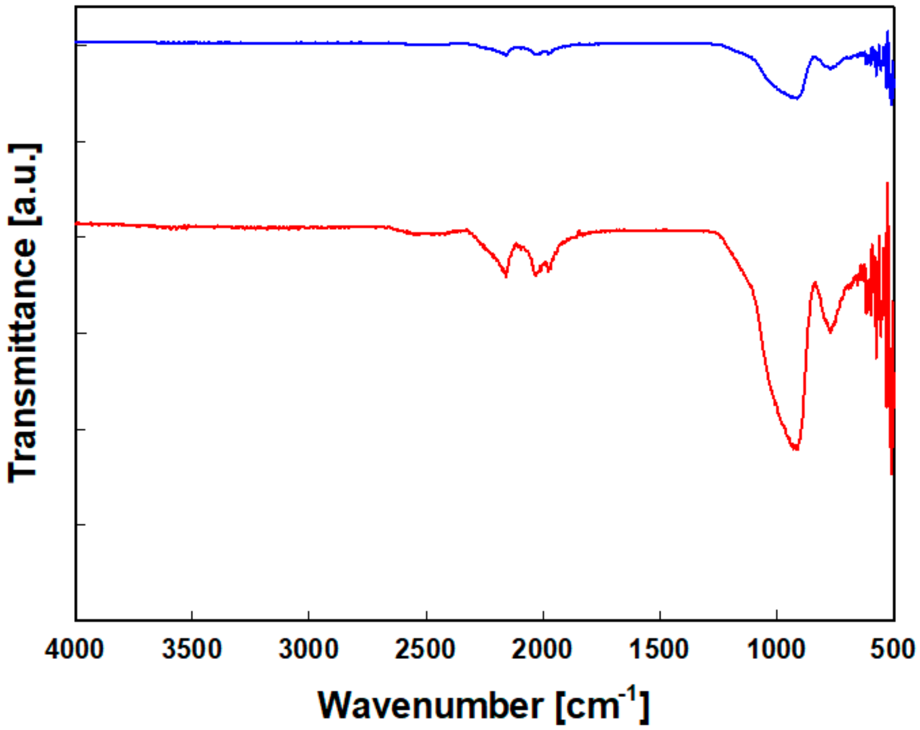

Figure S4. FT-IR spectra of Ag NWs network films after ethanol dipping process. (**blue**: thin Ag NWs network, **red**: thick Ag NWs network).

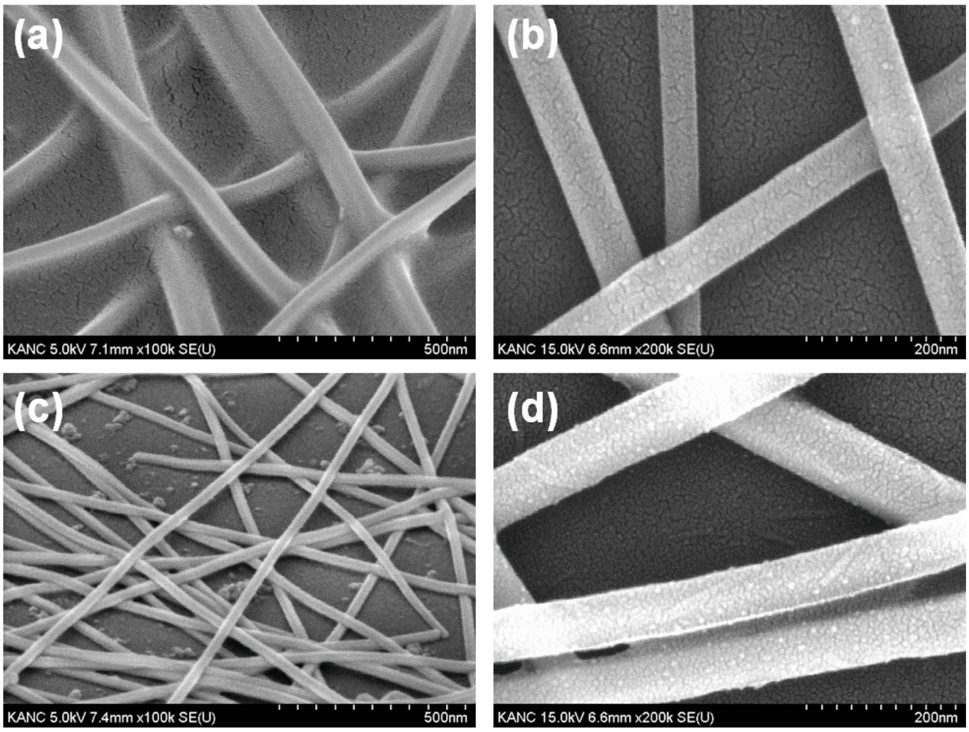

Figure S5. SEM images of thick Ag NWs network followed by different post-treatment. (**a**) SEM images of the pristine thin Ag NWs network, (**b**) thin Ag NWs network after thermal annealing at 130 °C for 20 min, (**c**) thin Ag NWs network after solvent dipping, and (**d**) thin Ag NWs after N_2 gas-blowing.

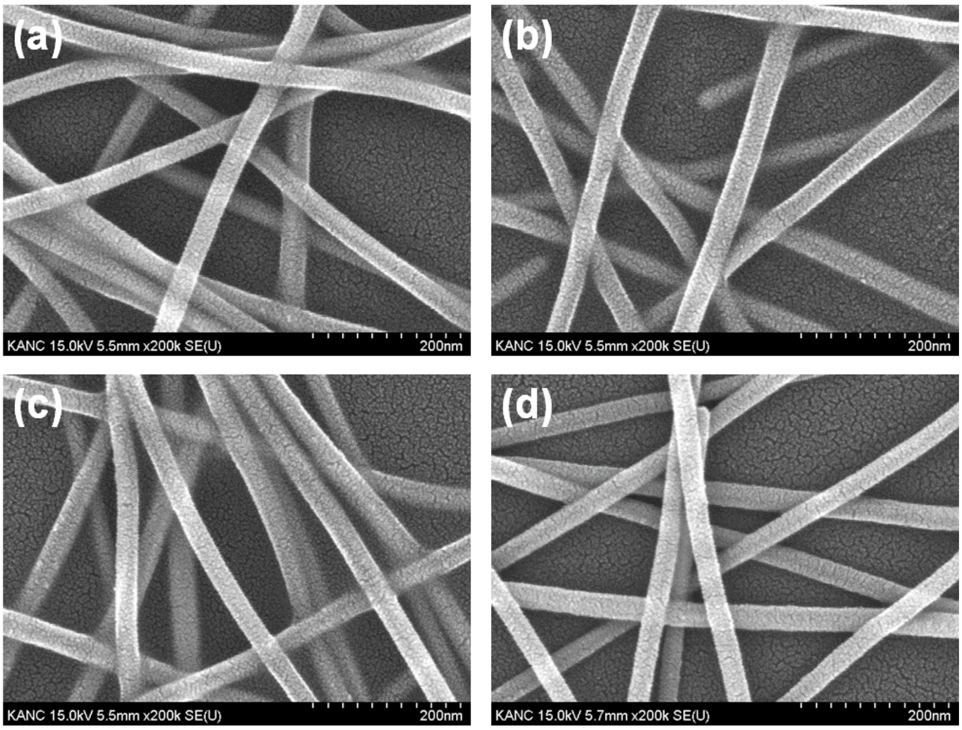

Figure S6. SEM images of thin Ag NWs network followed by different post-treatment. (**a**) SEM images of the pristine thin Ag NWs network, (**b**) thin Ag NWs network after thermal annealing at 130 °C for 20 min, (**c**) thin Ag NWs network after solvent dipping, and (**d**) thin Ag NWs after N_2 gas-blowing.

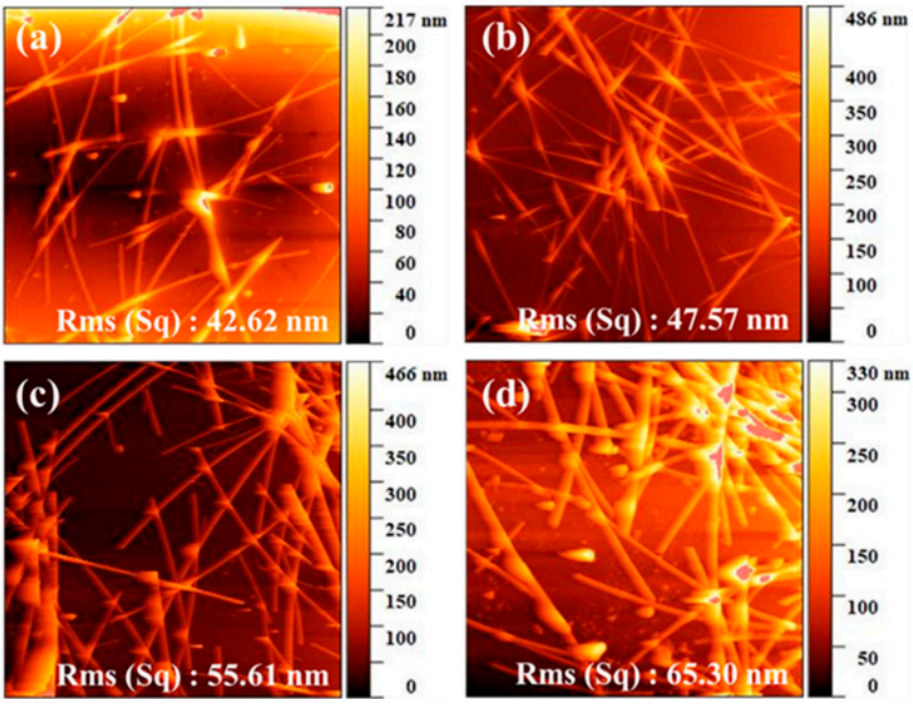

Figure S7. AFM topographic images of thick Ag NWs network. (**a**) Pristine Ag NWs network. (**b**) Ag NWs network after thermal annealing at 130 °C for 20 min. (**c**) Ag NWs network with solvent dipping. (**d**) Ag NWs network after N_2 gas-blowing.

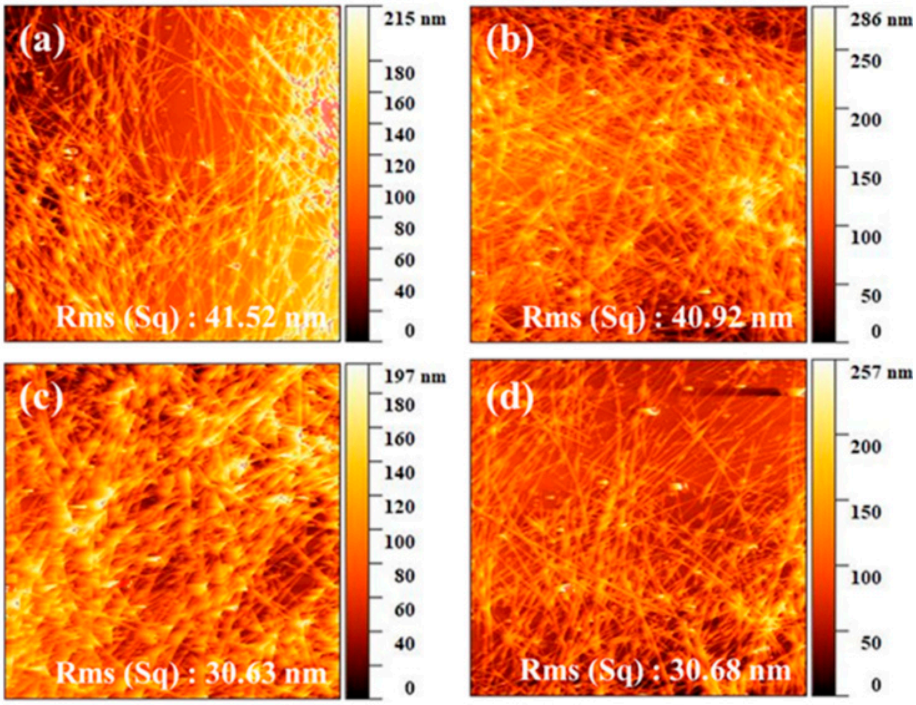

Figure S8. AFM topographic images of thin Ag NWs network. (**a**) Pristine Ag NWs network. (**b**) Ag NWs network after thermal annealing at 130 °C for 20 min. (**c**) Ag NWs network with solvent dipping. (**d**) Ag NWs network after N_2 gas-blowing.

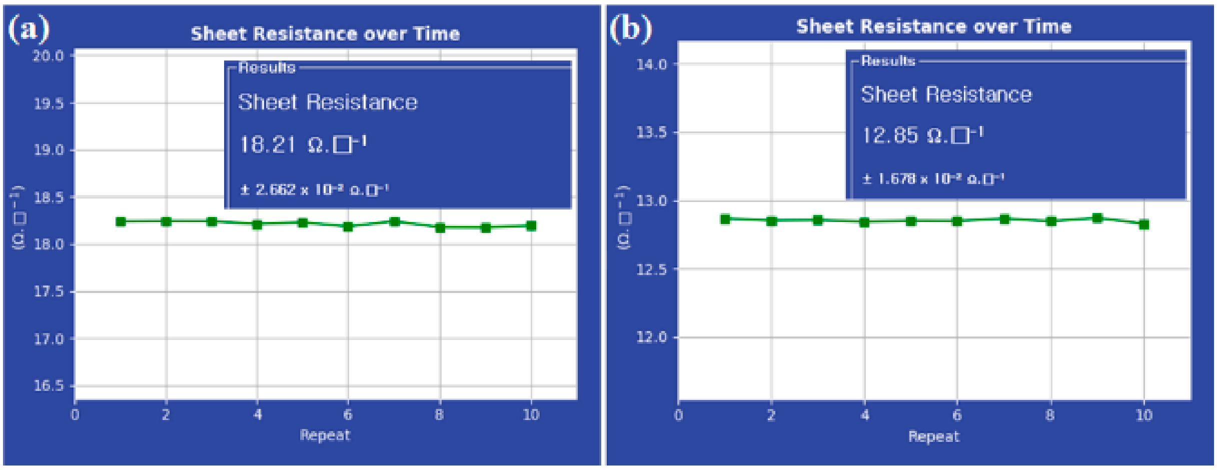

Figure S9. (a) Sheet resistance of Ag NWs network with thin diameter (a)and thick diameter (b).

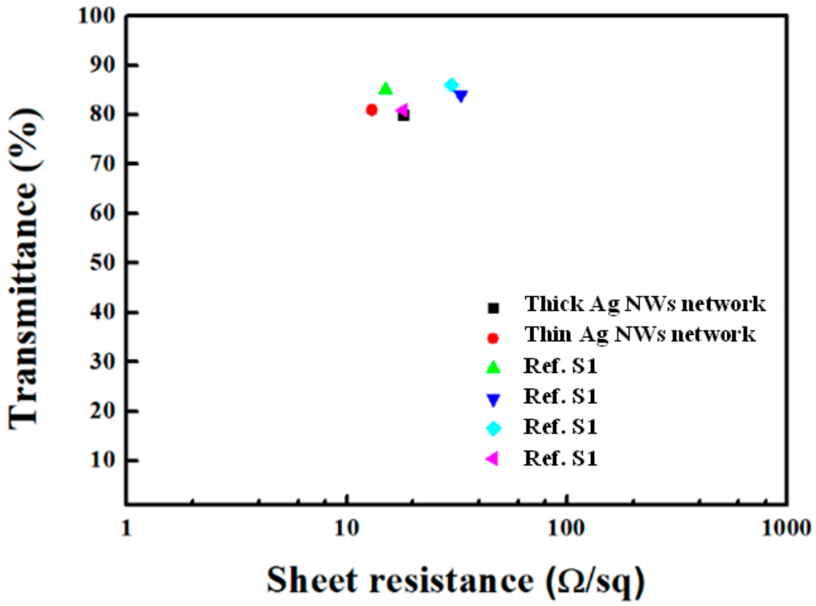

Figure S10. Sheet resistance versus transmittance from selected publications are also plotted for comparison.

Supplementary reference

S1. ACS Nano 2014, 8, 1590-1600.

S2. ACS Nano 2010, 4, 2955-2963.

S3. Nanoscale 2017, 9, 1978-1985.

S4. Nat. Nanotech. 2011, 6, 788
